# Inheritance of foliage color of common rosemallow (*Hibiscus moscheutos* (L.)) subspecific hybrids

**DOI:** 10.1186/s40529-019-0251-4

**Published:** 2019-03-12

**Authors:** Kaitlin Barrios, John M. Ruter

**Affiliations:** Department of Horticulture, University of Georgia, 1111 Miller Plant Science, Athens, GA 30602 USA

**Keywords:** Mendelian inheritance, Anthocyanin, Malvaceae, Intraspecific hybrid

## Abstract

**Background:**

Common rosemallow (*Hibiscus moscheutos* (L.)) is a native wetland perennial taxon that has been widely used as an ornamental landscape plant for several decades. Its showy blooms, attractive form and foliage, and hardiness attracted the attention of plant enthusiasts, leading to extensive hybridization and subsequent selection of superior genotypes. Red foliage color is a desirable trait, therefore we investigated the mode of inheritance of this trait in *H. moscheutos* subsp. *lasiocarpos* (Cavanilles) O. J. Blanchard with intraspecific hybrids of *H. moscheutos* subsp. *moscheutos* (L.).

**Results:**

Two red-foliaged seed parents of *Hibiscus moscheutos* subsp. *moscheutos* (L.) were crossed with green-foliaged paternal parent *H. moscheutos* subsp. *lasiocarpos*. Two *F*_2_ full-sib families (*n *= 192 and 238) were each found to fit a 3 red: 1 green segregation ratio for foliage color using a Chi square goodness-of-fit analysis. For further evaluation of this segregation pattern, each parent was selfed, as were two red-foliaged *F*_2_ plants. The two red-foliaged parents yielded the expected all-red progeny (*n* = 53 and 178, 1 red: 0 green) and the green-foliaged parent yielded 244 green and 6 red plants, fitting the expected 0 red: 1 green (*P *= 0.704) ratio. Additionally, progeny from the two red-foliaged *F*_2_ plants fit the expected 1 red: 0 green ratio (*n* = 135 and 120).

**Conclusions:**

Results indicate the appearance of red foliage, in any amount, in the two subspecies utilized and our hybrids of hibiscus to be controlled by a single locus with a dominant allele for red foliage. We propose the gene be called “green foliage” where the dominant allele, *G,* yields a red foliage phenotype. When the recessive allele, *g*, is present in the homozygous form, progeny consist of an all-green foliage phenotype for *Hibiscus moscheutos* (L.). Understanding the mode of inheritance of red-foliage phenotype in hibiscus would prove useful in further ornamental breeding work.

## Background

Common rosemallow (*Hibiscus moscheutos* (L.)) is a perennial shrub native to wetland areas of North America and appreciated for its showy white to pink flowers. The abundant, ephemeral and large blooms have garnered this species widespread adoption, and as a result cultivation and breeding since the early 19th century (Winters 1970). Along with four other North American hibiscus species, common rosemallow belongs to section *Muenchhusia* within the genus in the Malvaceae family, and is diploid (2n = 2x = 38) (Skovsted 1935; Small 2004; Wise and Menzel 1971). Common rosemallow has had several botanical names over time with the currently adopted taxonomy splitting it into two subspecies: *moscheutos* and *lasiocarpos* (Cavanilles) O. J. Blanchard (Blanchard 1977, 2008). The distinctions are based on the presence (subsp. *lasiocarpos*) or absence (subsp. *moscheutos*) of hairs on the adaxial leaf surface, capsules, and bracts of the involucel (epicalyx), as well as their geographic ranges (Flora of North America Editorial Committee 2015). Subspecies *moscheutos* is found in the wild from Ontario to New Hampshire, south to Florida and west to Texas. *Hibiscus moscheutos* subsp. *lasiocarpos* exists naturally from Indiana, south to Alabama, west to Texas, including the mid-western states of Kansas and Oklahoma, and with disjunct populations in Florida, New Mexico, and northern Mexico (Chihuahua) (Blanchard 2008). Although the subspecies’ ranges overlap, the Mississippi River serves as a general border with subsp. *moscheutos* found mostly to its east and subsp. *lasiocarpos* to its west (Flora of North America Editorial Committee 2015). Given these native ranges, *H. moscheutos* is hardy from the United States Department of Agriculture (USDA) zones 4a to 9b, hence its other common name of hardy hibiscus (Winters 1970). Plants typically sprout stems from underground storage structures in Georgia during March with flowers first appearing in May, peaking late June/early July, and blooming sporadically into August and September. Plants set fruit as dehiscent capsules into late summer, senesce in the fall (October/November), and remain dormant during the winter until emerging in spring. Leaf morphology can vary in shape (broadly lanceolate to triangular-ovate), leaf base (cuneate to cordate), lobing (3-lobed or unlobed), and margins (crenate to serrate) (Flora of North America Editorial Committee 2015). Plants can measure 0.9–2.4 m tall and their form is generally upright to rounded shrub, however, as a wetland native its shape can appear asymmetric, leggy or floppy when planted alone (Godfrey and Wooten 1981). Many cultivars and ornamental hybrids, intra- or interspecific, have improved traits, particularly for form, flower, and foliage color.

Inheritance of foliage color has been observed and reported in various genera and is useful information when breeding ornamentals, potentially saving time and resources. Red to purple foliage has been reported to follow monogenic Mendelian 3:1 inheritance as either a dominant or recessive trait. Red foliage is controlled by a dominant monogenic allele in ornamental coleus (Nguyen et al. 2008) and some woody plants, such as beech (Blinkenberg et al. 1958; Heinze and Geburek 1995) and birch (Hattemer et al. 1990). In other plants, red foliage is inherited in a single-locus recessive fashion; for example, with barberry (Cadic 1992), redbud (Roberts et al. 2015), and tutsan (Olsen et al. 2006). In other cases, inheritance of red foliage is reportedly controlled by complementary gene action, as in hazelnut (Smith and Mehlenbacher 1996; Thompson 1985) and flowering dogwood (Wadl et al. 2010); or by a single gene with incomplete allelic dominance as with the bronze foliage allele (*Rt*) in crabapple (Alston et al. 2000; Sampson and Cameron 1965).

*Hibiscus moscheutos* subsp. *moscheutos* (L.) exhibits red foliage to varying degrees (Stout 1917) and has been selected for and exploited in several cultivars: ‘Crown Jewels’ PP11,857, ‘Plum Crazy’ PP11,854, ‘Midnight Marvel’ PP24,079, and ‘Summer Storm’ PP20,443 to name a few (Falstad III 2009; Fleming and Zwetzig 2001a, b; Hurd 2013). *Hibiscus moscheutos* subsp. *lasiocarpos*, on the other hand, displays entirely green foliage. The objective of this study was to identify the mode of gene action and number of loci determining the presence of red foliage in our specimen of *H. moscheutos* subsp. *lasiocarpos* (Cavanilles) O. J. Blanchard and in our *Hibiscus moscheutos* (L.) hybrids.

## Methods

**Fig. 1:**
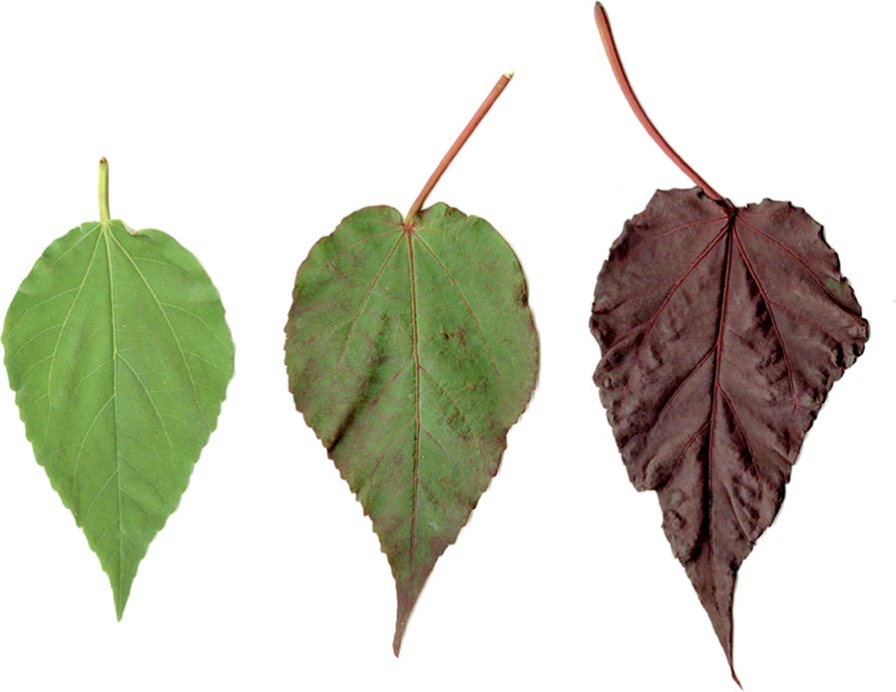
Leaves of hybrids of *Hibiscus moscheutos* L. showing foliage color ratings: 1 (left), 3 (middle), and 5 (right)

Plant material utilized for this study (from 2014 to 2017) was grown at the University of Georgia Durham Horticultural Farm in Watkinsville, GA. Two red-foliaged seed parents (R1 and R2) and one green-foliaged pollen parent (G) were used. R1 and R2 were intraspecific hybrids of *Hibiscus moscheutos* (L.) with red-foliaged cultivars in their background and were selected in 2013 as part of an ornamental breeding program. The pollen parent, G, (*Hibiscus moscheutos* subsp. *lasiocarpos* (Cavanilles) O. J. Blanchard) was obtained from Plant Delights Nursery (Raleigh, NC) in February of 2012. Two *F*_1_ populations (R1 × G and R2 × G) were generated and subsequently, two *F*_2_ populations were obtained from open-pollinations within each *F*_1_ population. The *F*_2_ plants were field-planted in June 2015 and a subjective rating on visual foliage color of the whole plant was given for each seedling in September 2015 using a scale of 1–5, where 1 = no red/entirely green, 2 = small amount of red, 3 = about 50% red, 4 = mostly red, and 5 = completely red (Fig. 1). Ratings were assigned based on the visual observation of the overall amount of red of the collective foliage in situ. Based on results from goodness-of-fit (Chi square) tests using observed *F*_2_ segregation ratios within families, testing of hypothesized gene action was implemented via selfing of P1s (R1 and R2), P2 (G), and 1 red-foliaged seedling from each *F*_2_ family. R1 and R2 had foliage color ratings of 5 and P2 (G) had a rating of 1. F2-R1 (an *F*_2_ seedling from R1 × G) had a rating of 5 and F2-R2 (an *F*_2_ seedling from R2 × G) had a rating of 2. Pollinations were performed in 2016 on plants in the field to obtain the following *S*_1_ populations: ⊗ R1, ⊗ R2, ⊗ G, ⊗ F2-R1, ⊗ F2-R2. Pollinations were done in summer 2016 in the mornings (7–9 a.m.) beginning June 24. A flower from the plant being selfed was removed and its pollen was spread onto flowers of the same plant, fully covering the stigmatic surfaces. Pollination tags were used to identify the fruits that were from controlled pollinations. Fruit were collected following dehiscence and seed were manually removed.

In March 2017, seed were scarified by soaking in 95–98% sulfuric acid (Avantor Performance Materials, LLC, Center Valley, PA) for 10 min and sown the following day in Jolly Gardener^®^ Pro-line C/L Growing Mix potting media (Old Castle Lawn and Garden, Pageland, SC) incorporated with Micromax^®^ micronutrients (Everris NA Inc., Dublin, OH) at 594 g m^−3^ in trays (26 × 50 × 6 cm) and kept under greenhouse conditions. Germinated seedlings were individually transplanted to 25-cell-pack tray filled with the same potting media as previous mixed with perlite (2:1, v/v) and were fertilized weekly with a liquid fertilizer at 200 mg L^−1^ (20–10–20, N–P_2_O_5_–K_2_O-Jack’s Professional^®^ J.R. Peters, Inc., Allentown, PA). After several weeks in the greenhouse, they were transplanted outside to 2.8 L nursery containers between May 17 and 26, 2017, top-dressed with 11 g pot^−1^ of controlled-release fertilizer (16–6–12, N–P_2_O_5_–K_2_O-Harrell’s Polyon™, Lakeland, FL) and placed outside on a ground cloth surface under regular overhead irrigation. Foliage color was rated for each plant once between July 24 and Aug. 2, 2017. Within each *S*_1_ family, the number of plants with a rating from 2 to 5 were pooled to obtain the observed red-foliaged value. Plants with a rating of 1 were considered green, or lacking red, foliaged (Fig. 1). Data from *F*_2_ families was used to propose hypotheses of single gene action for red foliage color in hardy hibiscus. Goodness-of-fit, or Chi square (χ^2^), tests were used to evaluate the fit of *F*_2_ and selfed populations to the proposed model. Chi square critical value used was ${\upchi^2}_{0.05,1} =3.841$ for the two observed phenotypes (Dowdy et al. 2004). P-values were calculated using the R program (R Core Team 2016).

## Results

Each of the two full-sib families of the *F*_2_ generation fit the phenotypic ratio of 3:1 for foliage color, where red is dominant to green (Table 1). *F*_2_ seedlings from Family 1 (R1 × G) and Family 2 (R2 × G) had Chi square values less than the critical value at 1.361 and 0.050, respectively. All *S*_1_ progeny from each of the red foliaged seed parents (R1 and R2) and a red-foliaged *F*_2_ seedling (F2-R1 and F2-R2) from each of the two families displayed red foliage (rating from 2 to 5), except for 2 plants out of 120 from ⊗ F2-R2. These *S*_1_ plants from ⊗ F2-R2, while having two individuals with green foliage, did not significantly differ from the predicted 1 red: 0 green ratio (*P* = 0.856). *S*_1_ progeny from the green-foliaged parent (G) resulted in 244 green-foliaged plants. Although 6 plants displayed some amount of red among ⊗ G progeny, the observed ratio was not significantly different from the expected 0 red:1 green ratio (*P *= 0.704).

**Table 1:** Number of observed and expected plants for foliage color with corresponding Chi square and *P* values, where ${\upchi^2}_{0.05,1} =3.84$

Parent(s)	Generation	Plants observed (no.)		Expected ratio	Plants expected (no.)		χ^2^	*P*
		Red (rating: 2–5)	Green (rating: 1)	Red:Green (rating: 2–5:1)	Red (rating: 2–5)	Green (rating: 1)		
R1 × G	*F*_2_	151	41	3:1	144	48	1.361	0.243
R2 × G	*F*_2_	180	58	3:1	179	60	0.050	0.823
⊗ R1	*S*_*1*_ from P ♀	53	0	1:0	53	0	–	–
⊗ R2	*S*_*1*_ from P ♀	178	0	1:0	178	0	–	–
⊗ G	*S*_*1*_ from P ♂	6	244	0:1	0	250	0.144	0.704
⊗ F2-R1	*S*_*1*_ from *F*_2_	135	0	1:0	135	0	–	–
⊗ F2-R2	*S*_*1*_ from *F*_2_	118	2	1:0	120	0	0.033	0.856

## Discussion

The results of these experiments indicate that red foliage phenotype in our hybrids of *Hibiscus moscheutos* (L.) is determined at a single locus. From Chi square analyses, we concluded that red foliage is completely dominant to the appearance of no red in foliage. When each of the parents with contrasting foliage colors was self-pollinated, the resultant progeny had the same foliage color as the parent from which they were selfed, suggesting homozygosity. Additionally, when a red-foliaged parent (genotype *GG*) was crossed with the green-foliaged parent (genotype *gg*) of both Family 1 and 2, *F*_*2*_ progeny displayed a 3 red: 1 green (genotype 1 *GG*: 2 *Gg*: 1 *gg*) segregation ratio for foliage color. The *S*_*1*_ progeny from the two *F*_*2*_ red seedlings also fit the 1 red: 0 green ratio, indicating homozygosity (genotype *GG*). We propose that the locus controlling the appearance of red, in any amount (rating 2–5), in the foliage of *Hibiscus moscheutos* (L.) be named “green foliage” with alleles *G* and *g*.

The observed deviations from the expected phenotypic ratios in progeny from ⊗ G and ⊗ F2-R2 are due to chance as they are statistically nonsignificant and could be explained by pollen contamination from pollinators or wind during controlled crosses or human error during the many steps of seed and plant handling. Other epistatic models were tested on the observed segregation ratios of *F*_*2*_ populations, such as dominant gene interaction (9:6:1) and duplicate gene action (15 red: 1 green), but no other model besides 3 red: 1 green was found to fit both *F*_*2*_ full-sib progeny. *F*_*2*_ progeny from R1 × P2 did fit dominant suppression gene action (13 red:3 green) (*P* = 0.355), however the *F*_*2*_ progeny from R2 × P2 did not, and progeny from selfing a red-foliaged *F*_*2*_ plant (⊗ F2-R1) did not display any progeny with all-green foliage which could be expected with an 13:3 epistatic inheritance model.

Investigation into the biochemical pathway leading to red foliage phenotype in common rosemallow is outside the objective of this study, and no investigation identifying anthocyanin production in *Hibiscus moscheutos* (L.) foliage has been found in current literature. In many angiosperms, red to purple appearance of vegetative and reproductive tissue is due to anthocyanins, however, the presence and concentration of other pigments, such as chlorophylls and carotenoids, can have a contributing effect on foliage coloration (Lee 2002; Taiz et al. 2015). Anthocyanins are a diverse class of pigments that can appear red, purple, pink or blue and belong to the flavonoids, a type of secondary metabolite (Taiz et al. 2015). Anthocyanins accumulate in the vacuole of the cell and have been shown via dissection analyses to reside in ground tissue (layer two; palisade and spongy mesophyll cells) of leaves rather than dermal tissue (layer one; epidermis) (Lee 2002; Lee and Collins 2001). Lightbourn et al. (2008) reported dark red/violet to black foliage of pepper (*Capsicum annuum* L.) had anthocyanins in the vacuoles of palisade mesophyll and spongy mesophyll cells whereas green foliage did not. In addition to the concentration of anthocyanins, the presence of carotenoids and chlorophylls can affect the visible color of foliage. Carotenoids are accessory pigments to chlorophyll during photosynthesis and are located in chloroplasts. There are several forms of carotenoids (β-carotene, lutein, violaxanthin, etc.) that can range in appearance from yellow, orange to red. While uncommon, a few cases of red foliage being attributed to carotenoids have been reported, such as in common box (*Buxus sempervirens*) by Ida et al. (1995), Lee (2002).

Foliage shade and hue can vary considerably since pigments, particularly anthocyanins, can be influenced by many abiotic and biotic factors. While colorful flowers and reproductive structures are commonly agreed on as advantageous to survival, the primary function of anthocyanins in foliar tissue is not widely agreed upon. Some theories that researchers have put forth were summarized by Santos-Buelga et al. (2010) and include: photoprotection, antioxidant activity, anti-herbivory, and oxidative signaling. Although the exact role(s) of anthocyanins in common rosemallow is currently undetermined, the occurrence of red foliage has resulted in preference and selection by consumers. The red intensity in University of Georgia hybrid hibiscus lines varies and people typically prefer darker or more intense red color foliage. Consumer personal feedback evaluation panels have consistently chosen plants with red foliage over plants with green foliage which is also reflected in many recent commercial releases.

Due to the range in the total amount of red pigmentation in the foliage of hybrids and the potential factors affecting anthocyanin and secondary pigment concentrations, we pooled all hibiscus with any amount of red pigmentation into one phenotypic grouping. Wadl et al. (2010) grouped hybrids similarly, with *Cornus florida* (L.), and determined red foliage color to be controlled by a completely dominant allele at a single locus. This type of inheritance of monogenic, dominant red over green foliage was also reported in *Betula pendula* var. ‘Purpurea’ by Hattemer et al. (1990), who observed a gradation (in their case) of purple foliage. Similar findings were reported in *Fagus sylvatica* (L.) by Blinkenberg et al. (1958) and Heinze and Geburek (1995) whereby foliage was described as “copper” color. Similar inheritance of a dominant red–purple foliar phenotype by a single gene was reported in two cultivars of tetraploid coleus (*Solenostemon scutellarioides* (L.) Codd) by Nguyen et al. (2008), however, the recessive trait was described as an orange–yellow phenotype rather than green foliage.

This study has not observed any linkage of phenotypic traits with red foliage color. Although the red-foliaged parents R1 and R2 have glabrous foliage compared to the green-foliaged parent, G, which has pubescent leaves, *F*_*2*_ hybrids display different possible combinations of foliage color and pubescence (data not shown) suggesting independence of gene activity.

## Conclusion

Common rosemallow is an attractive native plant known for its showy blooms and newer hybrids have incorporated stunning red–purple foliage. From crossing and selfing parents of contrasting foliage color, as well investigation of red foliage color in *F*_*2*_ plants, we found the appearance of red foliage, in any amount, to be controlled by a single locus with a dominant allele for red foliage. It follows that the green foliaged parent, a specimen of *Hibiscus moscheutos* subsp. *lasiocarpos* (Cavanilles) O. J. Blanchard, unless crossed with a red-foliaged hibiscus, should only yield all-green foliaged progeny. As red foliaged hibiscus is highly desirable in the nursery trade, ornamental breeders would benefit from understanding the type of inheritance of red foliage in *Hibiscus moscheutos* (L.) Additionally, an investigation into the genetic control of the range in intensity of red foliage would be worth exploring.

## Abbreviations

β-carotene: beta-carotene
F1: first generation from cross of P1 × P2
F2: second generation from cross of P1 × P2
G: green-foliaged *Hibiscus moscheutos* subsp. *lasiocarpos* (Cavanilles) O. J. Blanchard)
*G*: proposed dominant allele for red foliage phenotype
*g*: proposed recessive allele for red foliage phenotype
P1: seed/female parent of cross
P2: pollen/male parent of cross
R1 and R2: red-foliaged intraspecific hybrids of *Hibiscus moscheutos* (L.)
S1: first generation from selfing (cross with itself)
USDA: United States Department of Agriculture
⊗: crossing a plant using pollen of the same plant or genotype

## References

[CR1] Alston FH, Phillips KL, Evans KM. (2000) A *Malus* gene list. In: Proceedings of the Eucarpia symposium on fruit breeding and genetics, vols 1 and 2, International Society Horticultural Science, Leuven 1, pp 561–570

[CR2] Blanchard OJ Jr (1977) A revision of species segregated from *Hibiscus* sect. *Trionum* (Medicus) de Candolle sensu lato (Malvaceae). Dissertation, Cornell University

[CR3] Blanchard OJ (2008). Innovations in *Hibiscus* and *Kosteletzkya* (Malvaceae, Hibisceae). Novon.

[CR4] Blinkenberg C, Brix H, Schaffalitzky M, Vedel H (1958). Controlled pollinations in *Fagus*. Silvae Genet.

[CR5] Cadic A (1992). Breeding for ever-red barberries (*Berberis* spp.). Acta Hort.

[CR6] Dowdy S, Weardon S, Chilko D (2004). Statistics for research.

[CR7] Falstad CH III (2009) Hibiscus plant named ‘Summer Storm’. US Plant Patent 20,443, 27 Oct 2009

[CR8] Fleming DW, Zwetzig GA (2001a) Hibiscus plant named ‘Plum Crazy’. US Plant Patent 11,854, 1 May 2001

[CR9] Fleming DW, Zwetzig GA (2001b) Hibiscus plant named ‘Crown Jewels’. US Plant Patent 11,857, 1 May 2001

[CR10] Flora of North America Editorial Committee (2015). Magnoliophyta: Cucurbitaceae to Droseraceae.

[CR11] Godfrey RK, Wooten JW (1981). Malvaceae (Mallow Family). Aquatic and wetland plants of southeastern United States: dicotyledons.

[CR12] Hattemer HH, Steiner W, Kownatzki D (1990). Genetic markers in birch. Silvae Genet.

[CR13] Heinze B, Geburek T (1995). Searching for DNA markers linked to leaf colour in copper beech, *Fagus sylvatica* L. var. *atropunicea*. Silvae Genet.

[CR14] Hurd KA (2013) Hibiscus plant named ‘Midnight Marvel’. US Plant Patent 24,079, 10 Dec 2013

[CR15] Ida K, Masamoto K, Maoka T, Fujiwara Y, Takeda S, Hasegawa E (1995). The leaves of the common box, *Buxus sempervirens* (Buxaceae), become red as the level of a red carotenoid, anhydroeschscholtzxanthin, increases. J Plant Res.

[CR16] Lee DW, Gould KS, Lee DW (2002). Anthocyanins in leaves: distribution, phylogeny and development. Advances in botanical research incorporating advances in plant pathology.

[CR17] Lee DW, Collins TM (2001). Phylogenetic and ontogenetic influences on the distribution of anthocyanins and betacyanins in leaves of tropical plants. Int J Plant Sci.

[CR18] Lightbourn GJ, Griesbach RJ, Novotny JA, Clevidence BA, Rao DD, Stommel JR (2008). Effects of anthocyanin and carotenoid combinations on foliage and immature fruit color of *Capsicum annuum* L. J Hered.

[CR19] Nguyen P, Quesenberry K, Clark D (2008). Genetics of growth habit and development of new coleus (*Solenostemon scutellarioides* (L.) Codd) varieties with trailing habit and bright color. J Hered.

[CR20] Olsen RT, Ranney TG, Werner DJ (2006). Fertility and inheritance of variegated and purple foliage across a polyploid series in *Hypericum androsaemum* (L.). J Am Soc Hort Sci.

[CR21] R Core Team (2016) R: a language and environment for statistical computing. R Foundation for Statistical Computing, Vienna. http://www.R-project.org. Accessed 5 Oct 2018

[CR22] Roberts DJ, Werner DJ, Wadl PA, Trigiano RN (2015). Inheritance and allelism of morphological traits in eastern redbud (*Cercis canadensis* (L.)). Hort Res.

[CR23] Sampson DR, Cameron DF (1965). Inheritance of bronze foliage, extra petals and pendulous habit in ornamental crabapples. Proc Am Soc Hort Sci.

[CR24] Santos-Buelga C, Escribano-Bailon MT, Lattanzio V (2010). Recent advances in polyphenol research.

[CR25] Skovsted A (1935). Chromosome numbers in the Malvaceae. I J Genet.

[CR26] Small RL (2004). Phylogeny of Hibiscus sect. *Muenchhusia* (Malvaceae) based on chloroplast rpL16 and ndhF, and nuclear ITS and GBSSI sequences. Syst Bot.

[CR27] Smith DC, Mehlenbacher SA (1996). Inheritance of contorted growth in hazelnut. Euphytica.

[CR28] Stout AB (1917). Notes regarding variability of the rose mallows. Torreya.

[CR29] Taiz L, Zeiger E, Moller IM, Murphy A (2015). Plant physiology and development.

[CR30] Thompson MM (1985). Linkage of the incompatibility locus and red pigmentation genes in hazelnut. J Hered.

[CR31] Wadl PA, Wang XW, Pantalone VR, Trigiano RN (2010). Inheritance of red foliage in flowering dogwood (*Cornus florida* (L.)). Euphytica.

[CR32] Winters HF (1970). Our hardy hibiscus species as ornamentals. Econ Bot.

[CR33] Wise DA, Menzel MY (1971). Genetic affinities of North American species of *Hibiscus* sect. *Trionum*. Brittonia.

